# Combined Anticoagulation and Antiaggregation in Acute Cervical Artery Dissection

**DOI:** 10.3390/jcm10194580

**Published:** 2021-10-02

**Authors:** Philipp von Gottberg, Victoria Hellstern, Christina Wendl, Marc E. Wolf, Ludwig Niehaus, Hansjörg Bäzner, Hans Henkes

**Affiliations:** 1 Neuroradiologische Klinik, Klinikum Stuttgart, 70174 Stuttgart, Germany; v.hellstern@klinikum-stuttgart.de (V.H.); h.henkes@klinikum-stuttgart.de (H.H.); 2 Institut für Röntgendiagnostik, Zentrum für Neuroradiologie, Fakultät für Medizin der Universität Regensburg, 93053 Regensburg, Germany; christina.wendl@ukr.de; 3 Neurologische Klinik, Klinikum Stuttgart, 70174 Stuttgart, Germany; ma.wolf@klinikum-stuttgart.de (M.E.W.); h.baezner@klinikum-stuttgart.de (H.B.); 4 Neurologische Fachklinik, Rems-Murr-Kliniken, 71364 Winnenden, Germany; Ludwig.niehaus@rems-murr-kliniken.de; 5 Medizinische Fakultät, Universität Duisburg-Essen, 45147 Essen, Germany

**Keywords:** ischemic stroke, cervical artery dissection, anticoagulation, antiaggregation, vessel recanalization

## Abstract

Cervical artery dissection (CAD) is a frequent cause of stroke in young adults. Previous studies investigating the efficiency of anticoagulation (AC) versus antiplatelet therapy (AT) found an insignificant difference. We therefore retrospectively evaluated a combination of AC plus AT in patients with acute CAD regarding safety and efficacy. Twenty-eight patients with CAD and minor neurological symptoms/no major infarction received either single (*n* = 14) or dual AT (*n* = 14) combined with AC. Angiographic follow-up during hospitalization, 4-8 weeks and 3–6 months after CAD focused on occlusion, residual stenosis, and functional recanalization. Possible adverse events were surveyed. We compared the AC *plus* AT group to 22 patients with acute CAD treated with AC *or* AT. Compared to preceding AC-/AT-only studies, AC *plus* single or dual AT resulted in more frequent, faster recanalization. Frequency and severity of adverse events was comparable. No major adverse events or death occurred. Preceding works on conservative treatment of CAD are discussed and compared to this study. Considerations are given to pathophysiology and the dynamic of CAD. Combining AC *plus* AT in CAD may result in more reliable recanalization in a shorter time. The risk for adverse events appears similar to treatment with only AC or AT.

## 1. Introduction

A common cause of stroke in young and middle-aged adults is a spontaneous dissection of the cervical carotid or vertebral artery, which accounts for 10–25% of all strokes in patients with a mean age of 45 years [1,2,3,4,5]. Spontaneous cervical artery dissection (CAD) occurs at all ages; however, there is a slight dominance in males, and it occurs in females at a younger age [6].

Apart from the peak population, the annual incidence is estimated to be 0.97–1.5 per 100,000 for the vertebral artery and 2.5–3 per 100,000 for the carotid artery, respectively [4,7].

The primary risk factors for developing a carotid or vertebral artery dissection include current smoking (30–40%), dyslipidemia/hypercholesterolemia (19–40%), arterial hypertension (aHT, 25%), and a history of vascular disease (4%). Chiropractic maneuvers may also be a risk factor for carotid and vertebral artery dissection (30% in patients with vertebral artery dissection and 6% in carotid artery dissection) [8,9,10].

The untreated dissection of an internal carotid (ICA) or vertebral artery (VA) is associated with a 70–80% incidence of developing an ischemic stroke [8,11]. The recommended treatment from the current guidelines and major trials is based mainly on antiaggregation (AT) *or* anticoagulant (AC) medication with no superiority between them [12,13,14,15,16,17]. In a population with young patients who have sustained a minor ischemic stroke (or TIA or just local dissection), the bleeding risk is probably low, and a combined AC–AT strategy could be more effective than single therapy with either AC *or* AT.

We therefore retrospectively analyzed the combination of AC with single or dual AT therapy in 28 patients with acute internal carotid artery or vertebral artery dissection, to determine the time to recanalization onset and time to functional recanalization, and the incidence of adverse events such as cerebral hemorrhagic and ischemic events as well as extracranial hemorrhagic complications. The results were compared to previous studies using only AC *or* AT therapy.

## 2. Materials and Methods

### 2.1. Patients

Between 2009 and 2021, 684 patients (254 females) presented to our institution with an acute dissection of the cervical internal carotid or vertebral artery, diagnosed by ultrasound, computed tomography angiography (CTA), magnetic resonance imaging/angiography (MRI/MRA) and digital subtraction angiography (DSA), or any combination thereof. Brain imaging was also assessed to detect major infarction and/or hemorrhage at initial presentation. CADs were classified as acute if the symptom onset was within 5 days before presentation and matched the site of dissection. All initial and follow-up diagnostic imaging examinations were verified by board-certified neuroradiologists.

From this cohort, *n* = 611 patients received endovascular treatment consisting of ballon- and stent-assisted angioplasty; *n* = 23 patients received no vascular-related treatment due to advanced infarction and/or major intracranial hemorrhage. There were *n* = 50 patients with acute dissections of the cervical carotid or vertebral artery, and without an associated pseudoaneurysm who qualified for conservative management either due to minor neurological symptoms (NIHSS < 3, derived from clinical records) or endovascular non-accessibility with good collateralization (derived from initial angiographic studies).

### 2.2. Medication

Patients were randomly assigned to either of the two hospital departments dealing with neurovascular disorders (i.e., the department of neurology “I” or department of neuroradiology “II”). Different therapy regimens were chosen according to the discretion of the two senior authors. Patients managed through Department I received AC *or* AT (group I) as suggested by neurological guidelines. Patients under the responsibility of Department II received AC *plus* AT (group II).

Among the patients receiving AC *plus* AT treatment (group II), the drug combination was as follows:

- Certoparin/dabigatran etexilate or warfarin; acetylsalicylic acid (ASA) 100 mg/d (group IIa)
- Certoparin/dabigatran etexilate; ASA 100 mg/d and ticagrelor 180 mg/d (group IIb)
- Certoparin/dabigatran etexilate; ASA 100 mg/d and clopidogrel 75 mg/d (group IIc)

Certoparin was administered 1–6 weeks after the diagnosis, followed by dabigatran etexilate or warfarin. Anticoagulation was maintained until the last follow-up or if functional recanalization occurred within this period. It was left to the treating physician’s discretion to determine whether to switch to AT or stop drug therapy after 6 months if there was no functional recanalization.

AT therapy was kept single or dual until functional recanalization. In dual-drug AT, it was reduced to ASA 100 mg/d after the last follow-up if the vessel(s) remained occluded or stenotic at a rate > 59% following NASCET.

### 2.3. Follow-Up Examinations

Follow-up was performed through CT-/MR-brain-imaging, CT-angiographic imaging, MR-imaging or intra-arterial angiography at least twice within 10 days after the initial event, once within 4–8 weeks, and once within 3–6 months after the initial event. Imaging was also performed in case of recurrence or worsening of symptoms. The follow-up examinations were combined with a clinical examination, but upon the functional reopening of the dissected vessel(s), follow-ups were called off further.

Follow-up diagnostic imaging was based on gadolinium-based MR-angiography or digital subtraction angiography (DSA) of all supra-aortic arteries from a 4F transfemoral or transradial access site.

In a retrospective analysis, arteries without any antegrade perfusion on the site of dissection were considered occluded. A 50–99% reduction of the vessel diameter compared to a healthy, comparable segment of the same artery, or a comparable segment of the contralateral internal carotid artery or vertebral artery was considered a residual stenosis. A 0–49% lumen loss compared to a healthy, comparable segment of the same artery was considered a functional recanalization. Initially non-occluded arteries with a stenotic dissection were considered unaffected or residually stenotic if the recanalization grade of the stenosis was within 50–99% of a healthy distant, comparable artery segment ipsi- or contralateral.

### 2.4. Evaluation in Follow-Ups

In follow-ups, events were categorized as non-serious and major adverse events (NSAEs, MAEs). New, asymptomatic T2 or diffusion-weighted MRI lesions and any report of new, transient neurological symptoms that could be related to the dissected vessel(s) were rated as NSAEs. An MAE was defined as any incident of major bleeding according to the International Society on Thrombosis and Hemostasis [18]. Additionally, any new, permanent neurological symptom after therapy onset that could likely be caused by the dissected vessel(s) and any hemorrhagic event requiring medical treatment was considered an MAE.

### 2.5. Statistics 

Descriptive statistics were performed as mean ± standard deviation for continuous variables and counts and percentages (%) for categorical variables. To evaluate the treatment efficacy, a binary logistic model was constructed for each of the main outcomes, i.e., initial recanalization and functional recanalization. Group effects were expressed as odds ratios (OR) together with their 95% confidence intervals. Statistical significance was set at 5% (α). All statistical analyses were performed in R, version 4.0.2.

## 3. Results

### 3.1. Patients

A total of 90% of the enrolled patients showed minor neurological symptoms, and 10% had a good collateral status with endovascular non-accessibility of the dissected vessel.

In all groups, arterial hypertension was the most common risk factor, followed by dyslipidemia, current smoking, and a history of vascular disease and diabetes (Table 1.). 

Patients with newly diagnosed dyslipidemia or untreated/undertreated hypertension received statin treatment and antihypertensive treatment, respectively. The remaining patients were normotensive and normolipidemic during hospitalization, as were all patients at follow-ups. No new diabetes was diagnosed, and individual changes in smoking behavior were not studied.

The most common symptoms at initial presentation were minor neurological symptoms, mainly vertigo, head and neck pain, or dysarthria, comparable to the most common symptoms reported in larger studies. 

Initial MR-imaging showed embolic infarcts of minor to moderate volume connected to the supplying, dissected vessels in 52%, and no acute DWI lesions in 48% of all patients. One patient with spontaneous cervical left internal carotid artery dissection presented with a small subarachnoid hemorrhage due to an accompanying spontaneous extra- and intradural vertebral artery dissection. In this patient, only the carotid artery dissection was included in this study.

No patient suffered from hemiparesis or loss of consciousness at the initial presentation. 

### 3.2. Medication

A total of 21 females (16–67 years old, mean age 41 years) and 29 males (23–68 years old, mean age 48 years) received only AC (*n* = 4), or only AT (*n* = 18) (group I, *n* = 22), or a combination of AC plus single or dual AT (group II, *n* = 28).

In group II, subgroup IIa (AC *plus* ASA) included *n* = 14, subgroup IIb (AC *plus* ASA *plus* ticagrelor) included *n* = 11, and subgroup IIc (AC *plus* ASA *plus* clopidogrel) included *n*= 3.

In *n* = 26 dabigatran etexilate and *n* = 2 warfarin were administered following certoparin. 

### 3.3. Follow-Up Examinations and Evaluation

**Figure 1:**
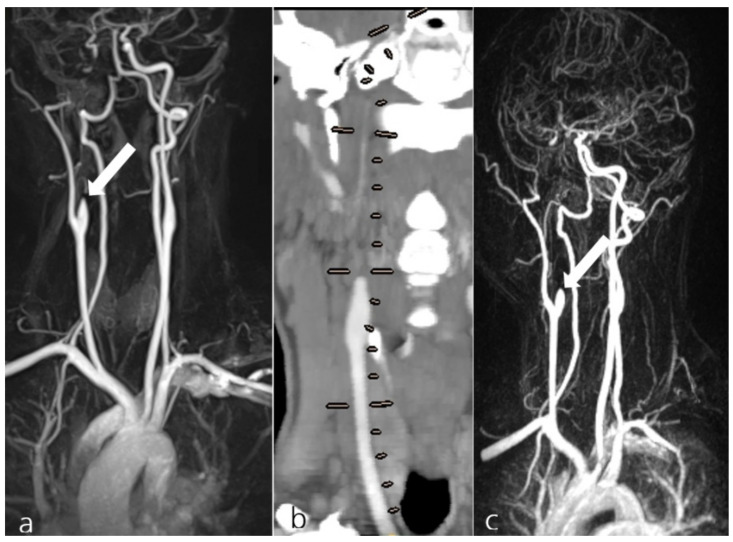
Exemplary display of patient #39 from group I, presenting with a spontaneous dissection of the right internal carotid artery (**a**, arrow). The MRA shows the collateral supply for the occluded artery. The patient received 100 mg ASA and 90 mg Prasugrel PO daily. Follow-up examinations at 4 years (CTA, **b**; lines show presumed course of the occluded right ICA) and at 4.5 years (**c**) showed the persistent occlusion of the dissected artery while still under AT treatment (**c**, arrow).

In group I (AC *or* AT, *n* = 22), occlusion or no change in stenosis, albeit treatment, was seen in 64%, and complete functional recanalization was achieved in 36% (8 out of 22). The time to initial changes in vessel occlusion or stenosis grade was 74 days (3–168), while the time to functional recanalization was 80 days (Figure 1).

**Figure 2:**
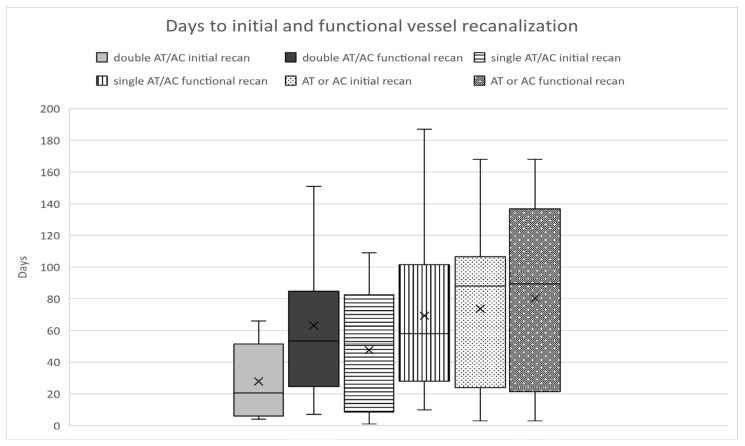
Days to the initial and functional vessel recanalization after cervical artery dissection in the different treatment groups.

In group II (AC *plus* AT), a persistent occlusion or no change in stenosis was seen in 29%, while 71% (20 out of 28) showed complete functional recanalization within 3–6 months after treatment onset. First changes in vessel occlusion or stenosis grade occurred after a mean time of 38 days and the mean time to functional recanalization was 66 days in total (Figure 2).

In group IIa (AC and single AT therapy), occlusion or no change in stenosis was seen in 29%, and complete functional recanalization was seen in 71% (10 out of 14). The time to initial changes in vessel occlusion/stenosis grade was 48 days (1–109); the time to functional recanalization was 69 days (means; 10–187, Figure 2). 

**Figure 3:**
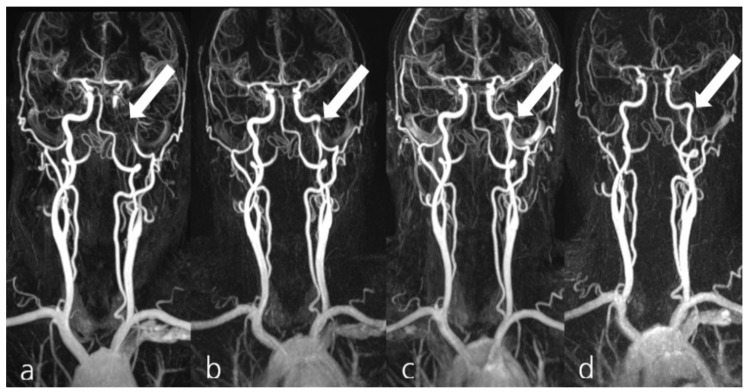
Exemplary display of patient #1 from group II, presenting with a spontaneous dissection of the left internal carotid artery (**a**, arrow). MRA confirmed the collateral supply for the occluded artery from the right-hand anterior circulation (**a**). The patient received 2× 150 mg dabigatran, 1× 100 mg ASA and 2× 90 mg ticagrelor PO daily. Follow-up MRA examinations 16 days (**b**), 29 days (**c**), and 141 days (**d**) demonstrated the progressive recanalization of the dissected artery (arrows).

In group IIb,c (AC *plus* ASA *plus* ticagrelor or clopidogrel), occlusion or no change in stenosis was seen in 29% and 71% showed complete functional recanalization (10 out of 14). The time to initial changes in vessel occlusion or stenosis grade was 28 days (4–66), while the time to functional recanalization was 63 days (Figure 3).

In group I (AC *or* AT), there was one MAE with an expanding groin hematoma requiring surgical intervention. Additionally, there were two NSAEs with TIAs and new DWI lesions in group I. Distribution of ICA and VA dissections was 16 ICA vs. 6 VA.

In group II, one patient in the AC *plus dual* AT subgroup suffered from transient speech impairment and sensory loss in the upper extremity ipsilateral to her internal carotid artery dissection 14 days after the initial event. She presented at our hospital immediately, and MR-imaging showed a small, new T2/DWI-lesion in the left temporal operculum. The symptoms resolved after a few hours. The former dissection-related stenosis, however, presented as a complete occlusion. This case was classified as an NSAE and recurrent stroke, representing 4% of group II (8% recurrent strokes in group IIb,c, respectively). The distribution of ICA and VA dissections was 22 ICA vs. 6 VA.

No other NSAE and no MAE was encountered in group II. Patients reported no other events at follow-ups. There were no reports of inguinal hematoma following catheter-based angiography that would have needed professional care apart from a usual treatment with a compression bandage.

### 3.4. Statistics

Comparison of both groups showed a significantly higher incidence and chance of initial and functional recanalization in the AC *plus* AT groups compared to AC or AT only. At the end of the 6-month interval, the incidence of initial recanalization was 1.75 times higher in group II compared to group I and 1.96 times higher for functional recanalization, respectively (*p*-value 0.041/0.021).

In addition, patients in group II had a 3.61 times higher chance for initial recanalization compared to group I and a 4.37 times higher chance for functional recanalization, respectively (*p*-value 0.033/0.015). Please refer to Tables S1–S5 in the supplementary materials to this study for complete statistics and the results in all 50 patients in detail.

## 4. Discussion

Current guidelines recommend AC or AT for the conservative management of cervical artery dissections. This is based on several AC- vs. AT-designed trials, following a concept with proven effectiveness and comparatively low risk. To the best of our knowledge, there are no reports in the literature regarding patients receiving combined AC *plus* AT treatment for spontaneous CAD. Beletsky et al. [19] (2003) reported five patients treated with an AC–AT combination out of *n* = 116 patients; however, the duration of treatment was not indicated, and there was no subgroup analysis.

To date, there is neither a recommendation nor dissuasion of an AC *plus* AT combination in the treatment of CAD in the literature.

Although the number of patients reported in this retrospective study is small, in terms of mean age, gender, and initial symptoms, it is comparable with larger prospective studies, such as TREAT-CAD [17] 2021 (*n* = 194) and CADISS [13] 2015/2019 (*n* = 250), as well as preceding major collective studies such as Beletsky et al. 2003 [19] (*n* = 116) and Georgiadis et al. 2009 [20] (*n* = 298).

The rate of major adverse events and complications in TREAT-CAD, CADISS, and Georgiadis et al. ranged from 0.5 to 3%. However, the definitions of an adverse event or major adverse event in these studies are not similar in detail. Major hemorrhage and other crucial elements of adverse event definitions in the mentioned studies nevertheless match the definition of an MAE in this study. Despite the more aggressive intervention in hemostasis in our group II, there were no MAEs.

The rate of recurrent stroke is 0.3–9% in preceding major studies (see Table 2), with 4%, the recurring stroke rate in our “AC *plus* AT” subgroup, being in a similar range.

When considering recurrent stroke incidents from a pathophysiological perspective, an uncontrolled recanalization of a former occluded, dissected vessel due to drug therapy might appear unfortunate. A clot, which may have formed in the distal lumen during the time of occlusion, could be washed out into the main branches of intracranial arteries, causing a major stroke. Table 2 demonstrates that a recurrent stroke is rare. Based on this infrequency, preceding studies such as CADISS could identify a stroke at presentation as a risk factor. Recanalization of former occluded vessels could not be connected to a higher risk of a recurrent stroke and other predecessors did not show such an association. The data of this study does not deviate either. Still, empirically and from the hemodynamic perspective, a faster recanalization seems to be favorable.

Among the 52% of patients presenting with acute DWI-lesions related to dissections, none in group I (AC *or* AT) presented with recurrent stroke. The only incidence of recurrent stroke in the AC/AT data was not based on an initial vessel occlusion followed by recanalization and had no DWI lesions on initial presentation.

The earliest possible recanalization of a closed or severely stenotic vessel by drug or interventional treatment, therefore, appears to be the most favorable to the authors, taking into account the pathophysiology of CAD and based on their clinical experience.

In the CADISS trial final results, the follow-ups have three vessel status definitions: vessel occlusion, partial recanalization in case of patent vessel lumen with residual narrowing to any extent, and complete recanalization as “the absence of any vessel abnormality” [13,15]. The cut-off between residual stenosis and functional recanalization in our study was set at 50% recanalization of the vessel lumen correlated with international guideline recommendations towards conservative vs. surgical/interventional treatment at 60% stenosis [21,22]. Therefore, the categories in CADISS and this study are not fully comparable. This study, however, excludes residual high-grade stenosis in the functional recanalization group that may have to be taken care of subsequently.

Considering this, the combination of AC *plus* AT in this study resulted in a rate of functional recanalization of vessels of 71.4% in total vs. the two groups “partial recanalization” (35.4%) and “full recanalization” (33.4%) equaling 67.4% in the CADISS final results. AC combined with dual AT resulted in an equal percentage as AC with single AT, which was 69.2% for both subgroups. Assuming that “partial recanalization” in CADISS does not exclude residual moderate and residual high-grade stenosis, we consider 71.4% functional recanalization as a superior result.

Our group I (AC *or* AT) was within the range of previous studies with a 36.4% functional recanalization.

In the subgroup analysis of mean time to functional recanalization, 63.1 days in group IIb,c (AC *plus dual* AT) vs. 69.3 days in group IIa (AC *plus single* AT) indicates an irrelevant difference.

When treating cervical artery dissections with AC/AT, the possibility of increasing the mural hematoma has been reported [23]. In this study, an increase in mural hematoma was observed in only one patient, as described in the results section. This patient received AC and dual AT as treatment, which would have been the worst combination if an increase of mural hematoma were associated with drug therapy. However, it was the only incidence among the whole study subgroup, with 13 more patients receiving dual AT and AC treatment. Furthermore, the effect of the observed hematoma increase was mild and not permanent, raising the question of whether such an effect must be of concern.

The CADISS 2019 final results reported 33.4% full recanalization and 35.4% partial recanalization after 90 days. The rate of 71.4% functional recanalization after a mean of 63.1 days in AC *plus dual* AT treatment in our study may represent a shorter time interval to reopening. However, these values are not comparable in detail since the CADISS study design did not require vessel status evaluation before the check-up at 90 days. In addition, no statement can be derived to benefit from a faster functional recanalization for the patient yet.

A limitation of this study is the lack of regular follow-up after functional recanalization of the previously dissected vessels. Possible silent re-stenosis or re-occlusions remain unnoticed. None of the patients in this study presented to our hospital with recurring or new symptoms related to the initial dissection after follow-up cancellation so far. This is also a limitation in the mentioned studies and could be solved only through long-term follow-ups. None of the preceding authors, however, assume a realistic risk of silent re-occlusions after a successful recanalization—nor do the authors of this study.

This retrospective study is further limited by the small number of enrolled patients. The allocation to group I or II was situational. The medication regimen in group I and II was not standardized. The timing of the follow-up examinations was not arranged according to a standard protocol and was merely triggered by clinical routine and individual circumstances.

In today’s clinical routine, there is significant uncertainty about which medicinal regimen for the treatment of cervical artery dissections combines the highest safety margins with optimal recanalization rates. Only a randomized, adequately powered trial can determine the benefits and safety of combined AC *plus* AT therapy in patients with CAD.

## 5. Conclusions

Based on our data, the combination of AC *plus* AT treatment of cervical artery dissections resulted in an equally low rate of adverse events and recurrent stroke than in previous AC- *or* AT-only studies. The rate of functional recanalization of dissected cervical arteries, however, was higher. The reopening of the dissected vessels to a non-stenotic state may be faster, especially with AC *plus dual* AT treatment.

A multicenter randomized controlled trial comparing AC, AT, and AC *plus dual* AT for the treatment of hemodynamically compensated cervical artery dissections appears warranted.

## Figures and Tables

**Table 1:** Prevalence of CAD risk factors in studies’ groups.

Risk Factor	Group I	Group IIa	Group IIb,c	Total
aHT	50%	36%	64%	52%
Dyslipidemia	23%	29%	29%	26%
Current smoking	27%	14%	7%	18%
History of vasc. disease	13%	7%	0%	8%
Diabetes	9%	0%	14%	8%

**Table 2:** Rate of recurrent stroke in preceding studies.

Authors/Study Name	Rate of Recurring Stroke	Time Interval
TREAT-CAD [17]	3.6%	3 months +/− 30 days
CADISS [13,15]	2.4%	12 months
Georgiadis et al. [20]	0.3%	3 months
Beletksy et al. [19]	9%	10 +/− 4 months

## Data Availability

The data presented in this study are openly available in www.mdpi.com/article/10.3390/jcm10194580/s1 at [doi], reference number [reference number].

## Supplementary Materials

The following are available online at www.mdpi.com/article/10.3390/jcm10194580/s1, Tables S1–S5 are supplementary materials connected to this study. Table S1: Main results Odds ratio for the occurrence of initial recanalization; Table S2: Main results Odds ratio for the occurrence of functional recanalization; Table S3: Main results Risk ratio for the occurrence of initial recanalization; Table S4: Main results Risk ratio for the occurrence of functional recanalization; Table S5: Summary of study results in detail.
