# Supplementary material for: Combined Anticoagulation and Antiaggregation in Acute Cervical Artery Dissection
Source: J Clin Med. 2021 Oct 2;10(19):4580. doi: 10.3390/jcm10194580 (PMC8509613; doi:10.3390/jcm10194580)
Supplement: Supplementary file 1 [file jcm-10-04580-s001.zip › jcm-1389745-supplementary.pdf]

Table S1. Main results Odds ratio for the occurrence of initial recanalization. Odds Ratio OR, Confidence Interval CI.

Characteristic	OR	95% CI	p-value
Grouping			
Group I	—	—	
Group II	3.61	1.14, 12.3	0.033

Table S2. Main results Odds ratio for the occurrence of functional recanalization. Odds Ratio OR, Confidence Interval CI.

Characteristic	OR	95% CI	p-value
Grouping			
Group I	—	—	
Group II	4.37	1.37, 15.2	0.015

[1] OR = Odds Ratio, CI = Confidence Interval

Table S3. Main results Risk ratio for the occurrence of initial recanalization. Risk Ratio RR, Confidence Interval CI.

Characteristic	RR	95% CI	p-value
Grouping			
Group I	—	—	
Group II	1.75	1.05, 3.00	0.041

Table S4. Main results Risk ratio for the occurrence of functional recanalization. Risk Ratio RR, Confidence Interval CI.

Characteristic	RR	95% CI	p-value
Grouping			
Group I	—	—	
Group II	1.96	1.15, 3.51	0.021

Table S5. Summary of study results in detail. Left/Right internal carotid artery LCI/RCI; left/right vertebral artery LVA/RVA; Certoparin Cert, Dabigatran Dabi, Acetylsalicylic Acid ASA, Prasugrel Pra, Clopidogrel Clo, Warfarin War; occluded/residual stenosis o/s.

#, Sex, age	Site of dissection	Stroke initially	Drug combination	Days to initial recanalization	Days to functional recanalization	NSAE/MAE
#1, M, 51	LCI	y	Cert/Dabi+ASA/Pra	16	141	
#2, M, 54	LCI	n	Cert/Dabi+ASA/Pra	51	51	
#3, F, 55	LCI	n	Cert/Dabi+ASA/Pra	6	56	
#4, M, 54	RCI	y	Cert/Dabi+ASA/Pra	66	66	
#5, M, 54	LCI	y	Cert/Dabi+ASA/Pra	7	7	
#6, F, 47	RCI	n	Cert/Dabi+ASA/Pra	6	65	
#7, F, 33	RCI	y	Cert/Dabi+ASA/Pra	o/s	o/s	
#8, M, 68	RVA	y	Cert/Dabi+ASA/Pra	4	24	
#9, F, 22	RVA	y	Cert/Dabi+ASA/Pra	o/s	o/s	
#10, M, 45	RVA	y	Cert/Dabi+ASA/Clo	o/s	o/s	
#11, M, 50	RCI	n	Cert/Dabi+ASA/Clo	25	25	
#12, F, 48	LCI	n	Cert/Dabi+ASA/Pra	o/s	o/s	NSAE
#13, M, 52	LCI	y	Cert/Dabi+ASA/Pra	45	45	
#14, F, 36	LCI	y	Cert/Dabi+ASA/Clo	53	151	
#15, M, 43	LCI	y	Cert/Dabi+ASA	77	77	
#16, M, 52	RVA	n	Cert/Dabi+ASA	o/s	o/s	
#17, F, 31	RCI	n	Cert/Dabi+ASA	109	109	
#18, M, 47	RCI	n	Cert/Dabi+ASA	10	10	
#19, F, 49	LCI	n	Cert/Dabi+ASA	51	51	
#20, M, 57	RCI	y	Cert/Dabi+ASA	o/s	o/s	
#21, M, 39	LCI	y	Cert/Dabi+ASA	99	99	
#22, M, 50	LVA	y	Cert/Dabi+ASA	o/s	o/s	
#23, F, 48	RCI	n	Cert/Dabi+ASA	64	64	
#24, M, 35	RCI	y	Cert/Dabi+ASA	o/s	o/s	

#25, F, 39	LCI, RCI	n	Cert/Dabi+ASA	10	34	
#26, F, 47	LCI	y	Cert/Dabi+ASA	4	187	
#27, M, 23	LVA	y	Cert/Dabi+ASA	52	52	
#28, F, 47	LCI	y	Cert/Dabi+ASA	4	187	
#29, M, 54	LCI	n	ASA	o/s	o/s	
#30, F, 16	RCI	n	ASA	o/s	o/s	
#31, F, 67	RCI	y	ASA/Pra	o/s	o/s	
#32, M, 43	RCI	n	ASA/Pra	19	19	
#33, M, 37	RCI	n	ASA/Pra	29	29	
#34, F, 41	RCI	y	ASA/Clo	o/s	o/s	
#35, M, 50	LCI, RCI	y	ASA/Pra	o/s	o/s	
#36, M, 59	LVA	n	ASA	o/s	o/s	
#37, M, 42	RCI	n	ASA	o/s	o/s	
#38, F, 40	LCI	n	ASA	o/s	o/s	
#39, F, 40	RCI	y	ASA/Pra	o/s	o/s	NSAE
#40, M, 31	RVA	n	ASA	94	94	
#41, M, 62	LCI	y	Cert/War	119	o/s	
#42, F, 42	LVA, RVA	y	Cert/War	o/s	o/s	
#43, M, 50	LCI	y	Cert/War	o/s	o/s	
#44, F, 43	LCI	n	ASA	91	91	
#45, F, 45	RCI	n	ASA	168	168	
#46, M, 61	RVA	n	ASA/Clo	o/s	o/s	
#47, F, 36	LCI	y	ASA/Clo	53	151	MAE
#48, F, 30	RCI, RVA	n	ASA/Pra	88	88	NSAE
#49, M, 56	LCI	y	Cert/War	3	3	
#50, M, 33	LVA	n	ASA	o/s	o/s	
